# Prevalent Variants in the LDLR Gene Impair Responsiveness to Rosuvastatin among Family Members of Patients with Premature Myocardial Infarction

**DOI:** 10.3390/jpm13121725

**Published:** 2023-12-18

**Authors:** Nguyen Trung Kien, Tran Tin Nghia, Nguyen Minh Hoang, Tran Nguyen Trong Phu, Pham Thi Ngoc Nga, Ha Thi Thao Mai, J. Luis Espinoza

**Affiliations:** 1 Faculty of Medicine, Can Tho University of Medicine and Pharmacy, Can Tho 900000, Vietnam; ntkien@ctump.edu.vn (N.T.K.); ttnghia@ctump.edu.vn (T.T.N.); httmai@ctump.edu.vn (H.T.T.M.); 2 Tra Vinh General Hospital, Tra Vinh 940000, Vietnam; 3 Faculty of Medicine, Chulalongkorn University, 1873, Rama 4 Road, Pathumwan, Bangkok 10330, Thailand; 4 Faculty of Health Sciences, Kanazawa University, Kanazawa 920-0942, Ishikawa, Japan

**Keywords:** LDLR gene variant, familial hypercholesterolemia, rosuvastatin, premature acute myocardial infarction, genetic metabolic disease

## Abstract

Background: Familial hypercholesterolemia (FH) is an inherited metabolic disorder characterized by high levels of low-density lipoprotein cholesterol (LDL-c) from birth. About 85% of all FH cases are caused by pathogenic variants in the LDLR gene. Individuals with FH have increased cardiovascular risk, including a high risk of premature myocardial infarction (PMI). Methods: We conducted an opportunistic exome screening to identify variants in the LDLR gene among Vietnamese patients with PMI treated at a general hospital in southern Vietnam. A cascade testing for LDLR variants was conducted in their relatives within three generations, and the effects of the LDLR variant on the response to rosuvastatin treatment were also studied using a comparative before-and-after study design on those who were eligible. Results: A total of 99 participants from the three generations of four PMI patients were recruited, mean age 37.3 ± 18.5 years, 56.6% males. Sanger sequencing revealed two variants in the LDLR gene: variant rs577934998 (c.664T>C), detected in 17 individuals within one family, and variant rs12710260 (c.1060+10G>C), found in 32 individuals (49.5%) in the other three families tested. Individuals harboring the variant c.664T>C had significantly higher baseline LDL-c and total cholesterol levels compared to those with variant c.1060+10G>C (classified as benign) or those without LDLR variants, and among the 47 patients subjected to a 3-month course of rosuvastatin therapy, those with variant c.664T>C had a significantly higher risk of not achieving the LDL-c target after the course of treatment compared to the c.1060+10G>C carriers. Conclusions: These findings provide evidence supporting the existence of pathogenic LDLR variants in Vietnamese patients with PMI and their relatives and may indicate the need for personalizing lipid-lowering therapies. Further studies are needed to delineate the extent and severity of the problem.

## 1. Introduction

Acute myocardial infarction (AMI) constitutes one of the leading causes of death and disability worldwide, and although it remains a disease prevalent in individuals of advanced age, in the last few years, a greater relative increase in the incidence of AMI among young and middle-aged individuals has been reported [1]. Despite older age, actively smoking, high blood pressure, diabetes mellitus, and total cholesterol and low-density lipoprotein (LDL-c) levels are the most prominent risk factors for AMI, in individuals with premature acute myocardial infarction (PMI), defined as AMI in men aged 18–55 years and women aged 18–65 years [2], other “non-traditional factors”, such as substance abuse, thrombophilia, immune disease, and psychological stressors have been implicated. In addition, genetic factors, particularly genetic alterations that alter the metabolism of cholesterol, have been shown to play an important role in the development of PMIs [3]. Indeed, it has been estimated that 63% of PMI can be attributed to heritability [3]. Familial hypercholesterolemia (FH) is an autosomal genetic disorder characterized by the presence of very high blood levels of LDL-c that can be manifested clinically by an early onset of atherosclerosis and cardiovascular disease. In about 85% of cases, FH is caused by pathogenic variants in the LDLR gene, with more than 3000 variants reported so far [4]. Variants in other genes that encode proteins involved in cholesterol metabolism, such as apolipoprotein B (ApoB) and proprotein convertase subtilisin kexin 9 (PCSK9), have also been reported. FH is an independent risk factor of PMI and those with complex variants (homozygous or compound heterozygous variant, etc.) may experience serious cardiovascular events as early as in their 20s [5].

Numerous studies have demonstrated the importance of early and appropriate management of FH to prevent the development of premature cardiovascular diseases. A large longitudinal cohort study on 3382 FH patients confirmed the importance of early identification and treatment of affected heterozygous individuals, where statins appear to benefit the primary prevention of fatal coronary disease [6]. In this regard, it has been estimated that 96–98% of deaths linked to cardiovascular disease in FH patients under the age of 40 could be prevented with statin therapy alone [7].

Nevertheless, despite the undisputable relevance of FH in the premature onset of cardiovascular disease, this condition is often underdiagnosed, and consequently, many people with FH are deprived of the benefits of early intervention. This is a major issue in low- and middle-income countries [8], where it has been estimated that less than 1% of FH patients are timely diagnosed [9]. This is also the case in Vietnam where early coronary artery disease was reported to be nearly 40% [10], yet studies on LDLR and PMI are still in their infancy. A seminal study conducted in Vietnam utilizing cascade screening identified a novel missense variant in FH patients [11]. A subsequent report from the Vietnam Familial Hypercholesterolemia Registry revealed that LDLR gene variants accounted for as many as 96.8% of FH patients. Notably, this study also discovered two novel LDLR variants that were considered unique to the Vietnamese population [12]. This raises concern about extrapolating the effectiveness of lipid-lowering therapy from foreign countries to Vietnam since the response to statin has been confirmed to be influenced by the genetic alterations in the LDLR gene [13,14,15].

Therefore, considering the scarcity of the available data, the potential difference in the LDLR variant pool, and the unknown response to lipid-lowering therapy in the Vietnamese population, we conducted an opportunistic LDLR variant screening in PMI patients along with cascade testing in their relatives. We further studied the effect of the LDLR variants on the response to rosuvastatin treatment using a before-and-after study design.

## 2. Patients and Methods

### 2.1. Subjects and Study Design

This prospective comparative study was conducted in a provincial hospital in southern Vietnam. We first recruited patients with confirmed PMI, who were diagnosed according to domestic guidelines and had an age of under 55 in males and 65 in females whose untreated LDL-c levels were of 155 mg/dL (4.0 mmol/L) or higher [16]. All the close members of the family of the recruited patients (parents, siblings, and children) were further recruited in a cascade screening manner. We excluded those who had advanced stages of kidney failure or cirrhosis, had secondary hyperlipidemia due to other conditions, such as nephrotic syndrome, hypothyroidism, hyperthyroidism, those who were on treatment with CYP3A4 inhibitors (ketoconazole, clarithromycin, erythromycin, etc.), or those who did not give consent. The study was conducted according to the principles of the Declaration of Helsinki of 1975, as revised in 2013 and was approved by the Institutional Review Board of Can Tho University (Approved on 31 March 2021, approval code #169/PCT-HDDD).

### 2.2. LDLR Gene Variant Screening and Patient Genotyping

PCR and sequencing primers were designed for analyzing the sequence of exons and exon-intron boundaries of the LDLR gene (Table S5). LDLR gene reference genomic sequence LDLR obtained from the National Center for Biotechnology Information Consensus CDS database (accession number NG_009060.1). Variants in the LDLR gene were determined in a molecular laboratory at Can Tho University of Medicine and Pharmacy, Vietnam. Genomic DNA was isolated from peripheral blood with EDTA anticoagulant (including those of the patient and members of their family) using the GeneJET Genomic DNA Purification Kit (Thermo Scientific, Waltham, MA, USA) following manufacturer guidelines and stored at −30 °C.

The 18 LDLR exons and exon-intron boundaries were amplified by primers synthesized by IDT (Integrated DNA Technologies) and listed in Table 1. PCRs (15 μL) contained 25–50 ng of genomic DNA, 0.5 U Taq Hot Start Polymerase (Takara Bio, Kusatsu, Japan), 0.1 μM each forward and reverse primers, 200 μM of each dNTP, and 1X PCR Buffer. The reactions were run in a SimpliAmp thermal cycler (Thermo Scientific, Waltham, MA, USA) with the annealing temperature LDLR set to 60 °C. The PCR products were analyzed on 1.5% agarose gel electrophoresis and then purified with the ExoSAP-IT reagent (Thermo Scientific, Waltham, MA, USA).

The amplicons were directly sequenced by the Sanger method using the BigdyeTM Terminator v3.1 Cycle Sequencing Kit (Applied Biosystems, Waltham, MA, USA) in both forward and reverse directions. The sequencing reactions were analyzed on an ABI 3500 Genetic Analyzer (Applied Biosystems, Waltham, MA, USA).

Sequencing results were analyzed with CLC Mainworkbench v5.5 software based on transcript version NM_000527.5, and nucleotides were counted from the first ATG translation initiation codon, calling variants according to T. den Dunnen nomenclature. To determine the pathogenicity of novel identified variants, functional prediction software packages and databases were used, including Polyphen-2: Polymorphism Phenotyping 2.0 (http://genetics.bwh.harvard.edu/pph2/ accessed on 10 December 2022), Mutation Taster (https://www.mutationtaster.org/ accessed on 10 December 2022), Clinvar (https://www.ncbi.nlm.nih.gov/clinvar/ accessed on 10 December 2022), and Varsome (https://varsome.com/ accessed on 10 December 2022).

### 2.3. Study Protocol

**Figure 1:**
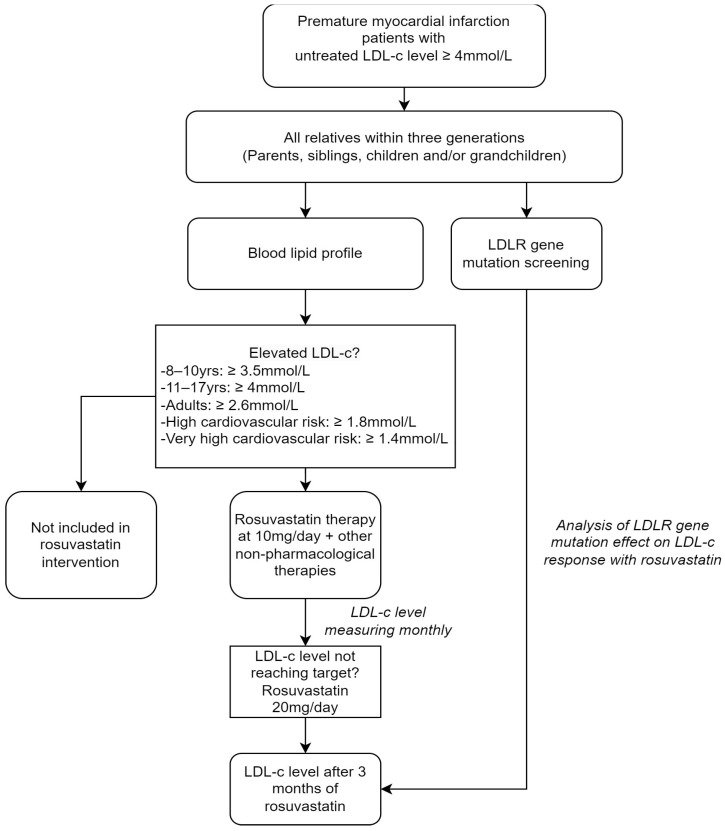
Study protocol. The rectangles indicate the steps conducted, with arrows showing the order of the steps. The number of participants is shown in each step.

The flowchart in Figure 1 depicts the outline of the study. All patients recruited, including PMI patients and their relatives within three generations (parents, siblings, children, and/or grandchildren), were assessed for their baseline demographics, medical history, cardiovascular risk factors, clinical symptoms, and blood lipid profile. Patients who had elevated LDL-c levels according to age and cardiovascular risk were further undergoing lipid-lowering therapy, including non-pharmacological management and rosuvastatin starting at 10 mg per day [16]. Non-pharmacological management encompassed patient consultations for a balanced diet, physical exercise, and smoking cessation. Rosuvastatin was given as a lipid-lowering therapy since this is the standard treatment utilized in Vietnam for patients with hypercholesterolemia, including those with FH. Patients were then followed monthly and LDL-c levels were evaluated. In the event of not reaching the LDL-c target, rosuvastatin was increased to a dose of 20 mg per day. After 3 months of lipid-lowering therapy, patients were re-evaluated for their lipid profile.

#### 2.3.1. Outcomes

The primary outcome of the intervention was the rate of achieving the LDL-c target after 3 months of intervention. The lipid-lowering intervention was termed achieved if the LDL-c level after 3 months was below the threshold of 4 mmol/L (8–10 years), 3.5 mmol/L (above 10 years), 1.4 mmol/L (adults with chronic coronary disease or diabetic patients with chronic complications), 2.5 mmol/L in adults, or LDL-c of less than 50% baseline level. Other outcomes of interest included the changes in LDL-c and cholesterol levels after 3 months of intervention and the incidence of rosuvastatin-associated side effects.

#### 2.3.2. Statistical Analysis

Normally distributed continuous variables were expressed as means and standard deviations, and as medians and inter-quartile ranges for skewed distributions. Categorical variables were expressed as counts and percentages. To compare the continuous variables between two independent groups, we used the Student’s *t*-test for comparing variables with a normal distribution or the Mann–Whitney U test for those with a skewed distribution. For subgroup analysis, the ANOVA F-test was used for three-group comparisons. All post-hoc pair-wise tests were conducted only after a significant F-test, which were adjusted for multiple testing by the Bonferroni method. Proportions were compared using the chi-squared test or Fisher’s exact test, where appropriate. The association between categorical variables was measured by the odds ratio. Multivariate logistic regression was used to study the effect of LDLR gene variants o″ the rate of LDL-c target achievement over the rosuvastatin treatment course. The covariates included in the multivariate model were age, sex, and baseline BMI. All analyses were performed with IBM SPSS 22.0 software. *p*-values below 0.05 were considered statistically significant.

## 3. Results

We identified four patients who presented PMI (Table S1) and a cascade screening of their relatives allowed us to recruit a total of 99 participants distributed in four families. The mean age of the participants was 37.3 ± 18.5 years, and males accounted for 56.6%. Obese and overweight participants made up 8.1% and 36.4%, respectively, while 31.3% of the subjects had an increased waist-to-hip ratio. Various cardiovascular risk factors were also identified, with a sedentary lifestyle being the most common (62.6%). Remarkably, eight individuals had a history of previous myocardial infarctions (Table 1). Regarding the blood lipid profiles, most participants had hyperlipidemia (71.7%), while increased LDL-c and high triglyceride levels were documented in 47.5% and 51.5% of participants, respectively. Mixed blood lipid disorder was not rare, with the combined increase in LDL-c and triglyceride levels being observed in 28.3% of participants (Table 1).

**Figure 2:**
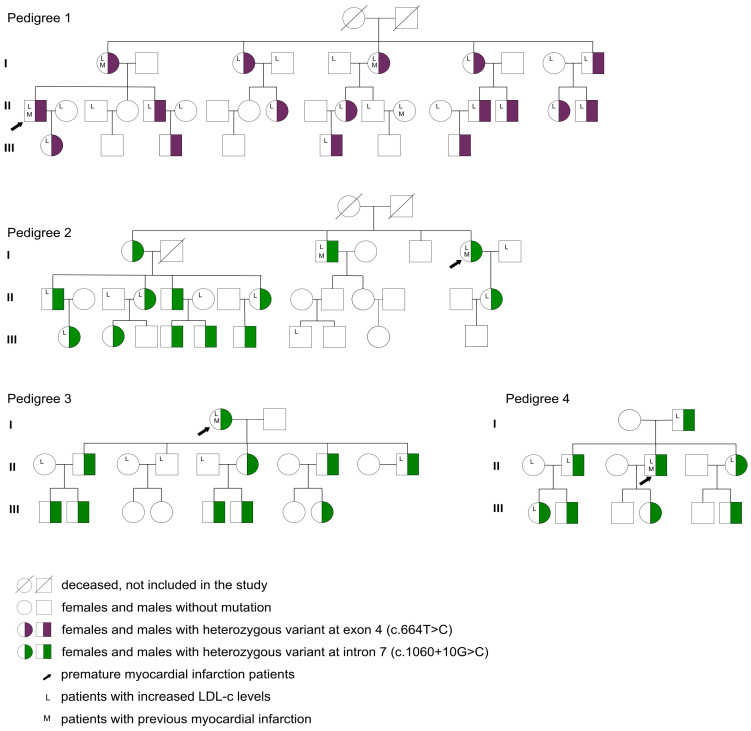
Pedigrees for families included in this study. The figure indicates the probands (black arrows) and other relatives spanning three generations (I, II, and III). Half-colored squares and circles stand for heterozygous variants in males and females, respectively. Blank squares and circles represent variant-negative participants. Letter L indicates patients with an increased LDL-c level according to age and cardiovascular risks. Letter M indicates those who had a previous myocardial infarction. LDL-c, low-density lipoprotein cholesterol.

Gene sequencing showed the presence of variants in the four patients with PMI (Table S2), and the cascade screening of their relatives (within three generations) revealed a prevalence of LDLR gene variants in 49.5% of them. We detected two variants: the variant rs577934998 (c.664T>C, at exon 4), which appeared in 17 patients in one family, and the variant rs12710260 (c.1060+10G>C, at intron 7) in 32 patients in the other three families (Figure 2 and Table S3). The variants seemed to affect patients regardless of their sex and appeared continuously across generations of the same bloodline (Figure 2). Among these patients, all had heterozygous genotypes (Table 1).

**Figure 3:**
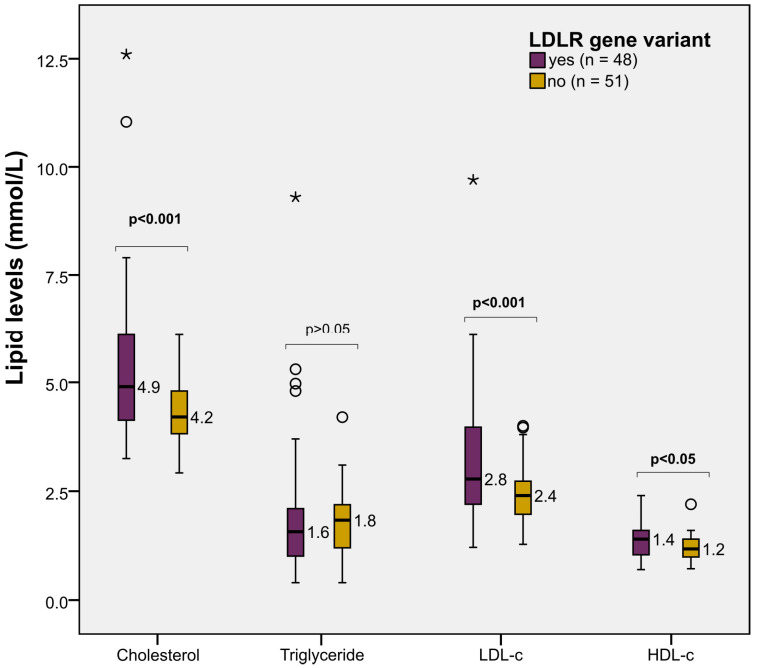
Lipid profile at baseline according to the LDLR gene variant status. Difference in the baseline levels of cholesterol components by LDLR gene variant status, including total cholesterol, triglyceride, LDL-c, and HDL-c levels in mmol/L. The boxplots indicate the distribution of lipid levels, with the medians in numbers. The blank circles and asterisks indicate mild and extreme outliers, respectively. All *p*-values were calculated by Student’s *t*-tests.

At baseline, patients who had LDLR gene variants had significantly higher levels of cholesterol and LDL-c than those without the variant (*p* < 0.001). However, triglyceride levels were comparable between the two groups, whereas HDL-c levels were slightly higher in patients who had the variants (*p* < 0.05) (Figure 3).

The frequency of LDLR gene variant was influenced by gender and was not associated with the rate of hypertension or diabetes. However, the presence of variants was significantly associated with the risk of LDL-c (OR 3.06, *p* = 0.007) and cholesterol (OR 5.79, *p* = 0.001) level elevation (Table 2).

**Figure 4:**
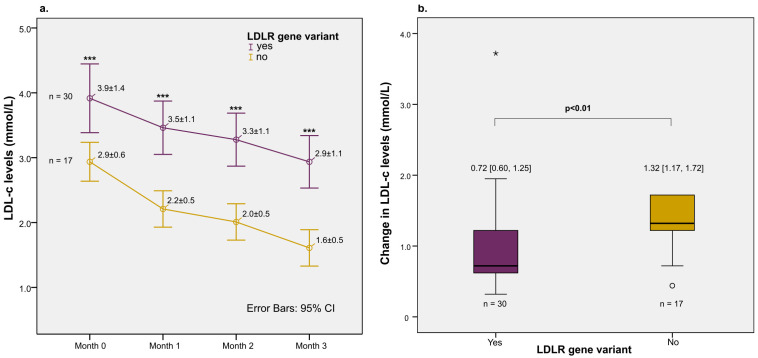
Effect of rosuvastatin treatment on LDL-c levels. (**a**) LDL-c levels in months 0, 1, 2, and 3 by LDLR gene variant groups (mmol/L). The circles and error bars indicate means and 95% confident intervals, respectively. The three consecutive asterisks indicate *p*-values below 0.001 in Student’s *t*-tests between two groups at each time point. (**b**) Change in LDL-c levels after 3 months by LDLR gene variant groups shown in boxplots. Numbers above each plot indicate medians and interquartile ranges. The *p*-value-value was calculated by the Mann–Whitney U test. The asterisks indicate extreme outli-ers.

There were 47 patients who met the criteria to be included in the lipid-lowering intervention, and all of them completed the 3-month treatment course (Table S4). Among them, 30 were LDLR gene variant-positive, and 17 were LDLR gene variant-negative. LDL-c levels consistently decreased over the course of treatment in both groups. However, the LDL-c levels remained significantly higher in the variant-positive group than those of the variant-negative group at each timepoint of follow-up (*p* < 0.001) (Figure 4a). After three months, patients with the LDLR gene variant had a delta change in LDL-c level of 0.72 [0.60, 1.25] mmol/L, which was significantly lower than the 1.32 achieved by the variant-negative group (*p* = 0.005) (Figure 4b).

After three months of rosuvastatin treatment, the LDL-c target level was achieved in 30 out of 47 patients. The LDLR gene variant accounted for 15 cases among target achievers and 15 among non-achievers [crude OR 0.13 (CI 95% 0.03–0.69), *p* = 0.02]. This suggests that the LDLR variants present in the studied population have functional attributes that affect the response to lipid-lowering therapies, namely that the variants may hinder the efficacy of lipid-lowering therapy and statin treatment. This modulation was confirmed in a multivariable logistic regression model, where the effect of the LDLR gene variant was adjusted for other confounders, including age, sex, and baseline BMI level (Table 3). No gastrointestinal symptoms, muscle pain, or any other adverse effects after rosuvastatin treatment were reported, and only transient laboratory test abnormalities, including two cases with AST elevation and three cases with ALT elevation, were documented.

**Figure 5:**
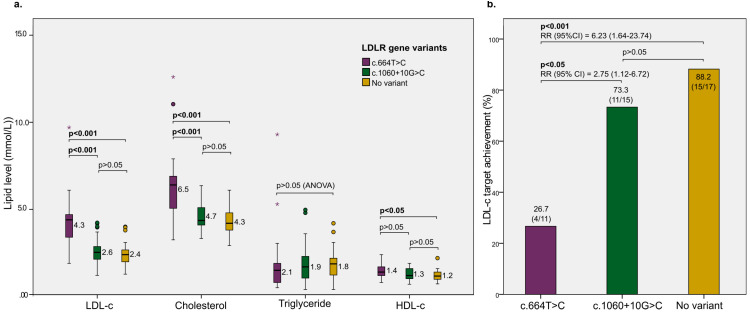
Subgroup analysis of the effect of LDLR gene variant types on lipid-lowering treatment. (**a**) The differences in the baseline levels of cholesterol components (total cholesterol, triglyceride, LDL-c, and HDL-c levels) according to the LDLR gene variant. Boxplots indicate the median distribution of lipid levels. The dots and asterisks indicate mild and extreme outliers, respectively. All other *p*-values were calculated by post-hoc pair-wise comparisons after the ANOVA F-test, adjusted by the Bonferroni method. (**b**) The difference in the rate of achieving the LDL-c target according to the variant status, with *p* values calculated using the chi-squared test and adjusted by the Bonferroni method. RR, risk ratio.

Finally, we conducted a subgroup analysis to investigate if the two variants in the *LDLR* gene differentially affect the patients’ responses to lipid-lowering therapy with rosuvastatin. Interestingly, patients with variant c.664T>C, which is classified as likely pathogenic, had significantly higher baseline LDL-c and total cholesterol levels compared to those with variant c.1060+10G>C (classified as benign) or those without variants (Figure 5a). Also, those with variant c.664T>C had a significantly higher risk of not achieving the LDL-c target after the course of lipid-lowering treatment compared to other LDLR gene variant types (Figure 5b).

## 4. Discussion

In this study, we have identified two prevalent genetic variants in the LDLR gene associated with FH in four Vietnamese patients who suffered PMI and in a high proportion of their close family members. A further interventional analysis suggested that one of the LDLR variants present in the studied population impairs the response to lipid-lowering therapy with rosuvastatin.

The variant c.1060+10G>C was detected in three families and the variant c.664T>C was found in one family. Variant c.664T>C is a missense or a single nucleotide variation (SNV), which results in an amino acid substitution (C222R) in the LDLR protein. Conflicting interpretations on the pathogenicity and clinical significance of this variant have been reported so far, as illustrated on the ClinVar and dbSNP databases [17]. In a cohort of 750 Taiwanese patients, only one case of the c.664T>C variant was reported [18], while in a previous study conducted in Vietnam, this variant was found in five out of 26 children tested [19]. The pathogenic mechanism of the c.664T>C variant is yet to be elucidated; however, its functional relevance could be similar to other variants occurring in exon 4 of the LDLR gene. Notably, exon 4 is the longest exon in LDLR gene and has the highest variant rate compared to the others, with numerous variants reported across the world [19,20,21,22]. While exon 4 along with exons 5 and 7 encode the ligand-binding domain of the LDLR protein, exon 4 encodes the critical ligand-binding section of the protein, thus any changes in the sequence may affect the folding of the protein thereby altering its functionality. Indeed, missense variants in this exon have been reported to reduce the LDLR receptor’s affinity to other cholesterol-associated proteins, such as APOE and APOB [23,24].

FH and familial combined hyperlipidemia (FCHL) account for an important proportion of premature atherosclerotic cardiovascular disease (ASCVD). In a previous study of 102 consecutive Viennese patients younger than 40 years who suffered an AMI, approximately 50% of cases were attributed to FH or FCHL [25]. Patients with premature coronary artery disease have a 15-fold higher frequency of FH variants than healthy controls [26]. In our study, all four PMI patients had a variant in the LDLR gene. Although the total number of screened subjected in this study was small, our data may suggest an alarmingly high prevalence of the phenomenon among Vietnamese PMI patients and prompt larger population-based screening.

The variant c.1060+10G>C is a splice site variant that appears to alter the mRNA processing in donor sites [27,28,29]. It has been proposed that this variant may reduce LDLR expression through competition between the aberrant donor splice and the original donor site [29]; this may explain the presence of a mild FH phenotype among heterozygous carriers of this variant. Interestingly, the c.1060+10G>C variant was the most frequent genetic defect (59%) in Brazilian patients with FH [28]. In our study, variant c.1060+10G>C was detected in 65.3% of the subjects tested, which may indirectly suggest the prevalence of this defect in Vietnamese patients.

In our primary analyses, we observed that variant-positive patients exhibited significantly elevated LDL-c levels and a poorer response to a 3-month regimen of lipid-lowering therapy and statin treatments. However, it is crucial to note that the primary contributor to this difference is very likely the c.664T>C variant, rather than the c.1060+10G>C variant. This can be seen in our subgroup analyses (Figure 5), where significantly higher baseline LDL-c levels and a lower rate of LDL-c target achievement were documented among c.664T>C carriers, not the c.1060+10G>C carriers, as compared to the variant-negative group. Our findings should contribute to the current classification of c.664T>C as a pathogenic variant and of c.1060+10G>C as a benign variant.

According to the UCL LDLR gene variant database, more than 3500 LDLR variants have been reported so far, with 77% of them being substitutions, 16% deletions, and 5% duplicates [30], although the pathogenicity of many of these LDLR variants remains unknown. Some studies have found that variants in the LDLR gene influence the response to statin [13,14,15]. One study from Brazil showed that the presence of LDLR variants was independently associated with higher odds of not achieving the LDL-C cut-off [31]. Of note, three c.1060+10G>C variant carriers had PMI (Figure 2) despite the variant’s benign classification. Many factors other than genetics have been proposed as risks for PMI, including male sex, lifestyle-related factors (smoking, alcohol use), hypertension, and diabetes [2]. Among the reported characteristics of these three patients, one had a history of smoking, and one presented with hypertension (Table S1), potentially elevating their susceptibility to PMI. However, this hypothesis should be considered in the context of the fact that variants in other genes pertinent to LDL-C metabolism were not explored in our study.

There are some limitations associated with this work. First, due to the limited funding, we could sequence only the LDLR gene using the Sanger technique. This approach may overlook other important variants in cholesterol metabolism-related genes such as LDLR, APOB, or PCSK9, and thus the causal relationship between the detected variants in LDLR genes and LDL-c levels might be incomplete. We were also unable to conduct a detailed family history assessment of the patients with PMI reported in the study, which could have provided a more comprehensive picture of the potential clinical impact of the LDLR variants reported here. In addition, the effect on the LDL receptor residual activity of the variants reported in this study is currently unknown, hindering the analysis of the genotype-variation effect of the response to statin treatment. Nevertheless, our work represents one of a few genetic studies on LDLR variants in PMI patients in Vietnam. In conclusion, a cascade genetic screening of the LDLR gene in the families of four Vietnamese PMI patients revealed two-point variants at exon 4 and intron 7, with one of them (the c.664T>C variant) being significantly associated with the increased LDL-c levels. A comparative study of the LDL-c level before and after a 3-month course of lipid-lowering intervention evidenced a lower rate of LDL-c target achievement in the c.664T>C variant variant-positive group, which points toward the use of more intensive pharmacological intervention in patients with confirmed LDLR variants. The prevalence of these variants among PMI patients’ relatives and its apparent influence on the response to statin treatment warrant larger studies with more thorough screening methods in the Vietnamese population or in those populations where the variants described here are prevalent.

## Figures and Tables

**Table 1:** Characteristics of the included participants.

Patient’s Characteristics	Number of Cases (%)
Age (years)	
≥60	14 (14.1%)
50–59	9 (9.1%)
40–49	25 (25.3%)
30–39	20 (20.2%)
20–29	5 (5.1%)
<20	26 (26.3%)
Mean age (years)	37.3 ± 18.5 *
Male sex (yes)	56 (56.6%)
BMI classification	
Obese (yes)	8 (8.1%)
Overweight (yes)	36 (36.4%)
Normal (yes)	55 (55.6%)
Increased waist length (yes)	19 (19.2%)
Increased waist to hip ratio (Yes)	31 (31.3%)
Cardiovascular risk factors	
Smoking (yes)	11 (11.1%)
Sedentary lifestyle (yes)	62 (62.6%)
Hypertension (yes)	10 (10.1%)
Diabetes (yes)	7 (7.1%)
Previous myocardial infarction (yes)	8 (8.1%)
Lipid profile	
Hyperlipidemia (yes)	71 (71.7%)
Increased LDL-c levels (yes)	47 (47.5%)
Increased triglyceride levels (yes)	51 (51.5%)
Increased cholesterol levels (yes)	24 (24.3%)
Decreased HDL-C levels (yes)	22 (22.2%)
Increased LDL-c + triglyceride levels (yes)	28 (28.3%)
Increased LDL-c + decreased HDL-c levels (yes)	12 (12.1%)
Increased LDL-c + triglyceride + decreased HDL-c levels (yes)	12 (12.1%)
LDLR gene variant (yes)	49/99 (49.5%)
Heterozygous genotype (yes)	49/49 (100%)
Exon 4 (c.664T>C) (yes)	17/49 (34.7%)
Intron 7 (c.1060+10G>C) (yes)	32/49 (65.3%)
Patients enrolled in rosuvastatin intervention (yes)	47 (47.5%)
Patients achieving LDL-c target after 3 months (yes)	30/47 (63.8%)

* mean ± standard deviation.

**Table 2:** Association between LDLR gene variants and patient’s characteristics.

Characteristics	LDLR Gene Variant		OR (CI 95%)	*p*-Value; χ2
	Yes	No		
Male sex (Yes)	28	28	1.04 (0.47–2.32)	0.91; 0.013
Increased LDL-c level (Yes)	30	17	3.06 (1.35–6.96)	0.007; 7.356
Increased cholesterol level (Yes)	19	5	5.79 (1.92–16.92)	0.001; 11.15
Hypertension (Yes)	6	4	1.6 (0.42–6.07)	0.357; 0.494
Diabetes (Yes)	5	2	2.72 (0.50–14.78)	0.210; 1.492

**Table 3:** Effect of LDLR gene variant on LDL-c target achievement.

Covariates	LDL-c Target After 3 Months		Crude OR (CI 95%)	Crude *p*-Value	Adjusted OR (CI 95%)	Adjusted *p*-Value
	Achieved	Not Achieved				
LDLR gene variant (yes)	15/30	15/17	0.13 (0.03–0.69)	0.02	0.13 (0.02–0.68)	0.02
Sex (females)	-	-	-	-	0.33 (0.07–1.52)	0.15
Age (+1 year)	-	-	-	-	1.02 (0.97–1.07)	0.42
BMI (+1 kg/m^2^)	-	-	-	-	0.99 (0.64–1.52)	0.95

## Data Availability

The datasets used and/or analyzed during the current study are available from the corresponding author on reasonable request.

## Supplementary Materials

The following supporting information can be downloaded at: https://www.mdpi.com/article/10.3390/jpm13121725/s1, Table S1: Characteristics of patients with premature MI. Table S2: Summary of the gene variants (variants) found in this study. Table S3: Baseline characteristics of patients with LDLR gene variant. Table S4: Characteristics of the patients included in the lipid-lowering cohort. Table S5: Primer sequences for PCR amplification.

## Abbreviations

T2DM: Type 2 diabetes mellitus; PCSK9: Proprotein convertase subtilisin/kexin type 9; SNPs: Single nucleotide polymorphisms; SBP: Systolic blood pressure; DBP: Diastolic blood pressure; UA: Uric acid; FBG: Fasting blood glucose; NGT: Normal glucose tolerance; mild: Improved multiple ligase detection reaction; SREBP-1c: Sterol regulatory element-binding protein-1c; BMI: Body mass index; Cr: Creatinine; BUN: Blood urea nitro-gen; GLU: Glucose; TG: Triglyceride; TC: Total cholesterol; HDL-C: High-density lipoprotein cholesterol; LDL-C: Low-density lipoprotein cholesterol; LDLR: Low-density lipoprotein receptor.
