# Supplementary material for: Prevalent Variants in the LDLR Gene Impair Responsiveness to Rosuvastatin among Family Members of Patients with Premature Myocardial Infarction
Source: J Pers Med. 2023 Dec 18;13(12):1725. doi: 10.3390/jpm13121725 (PMC10744399; doi:10.3390/jpm13121725)
Supplement: Supplementary file 1 [file jpm-13-01725-s001.zip › jpm-2684901-supplementary.pdf]

Supplementary tables.

Table S1. Characteristics of patients with premature MI

**Patient**	**No1**	**No2**	**No3**	**No4**
Pedegree	1	2	3	4
Age	45	55	57	47
Sex	1	2	2	1
Ethnic group	1	1	1	1
Living area	Countryside	Countryside	Countryside	Countryside
Job	Farmer	Farmer	Farmer	Farmer
Height (metre)	169	158	156	176
Weight (kilogram)	66	60	60	80
Waist (cm)	86	77	88	89
Hip (cm)	91	92	90	100
BMI (kg/m2)	23.11	24.03	24.65	25.83
History of smoking	2	2	2	1
Sedentary lifestyle	1	0	0	0
Hypertension	Yes	No	Yes	No
Blood pressure	140/90	120/80	140/90	130/80
Diabetes	No	No	No	No
History of MI	Yes	Yes	Yes	Yes
Glucose (mmol/L)	6.80	6.00	12.69	4.97
HbA1c (%)	5.60	5.80	6.10	5.20
Ure (mmol/L)	6.50	7.12	5.80	6.20
Creatinin (mcmol/L)	96.00	99.08	87.30	112.30
eGFR (mL/min/1.73m2)	86.00	98.00	63.52	66.90
SGOT (U/L)	29.00	17.00	38.00	128.00
SGAT (U/L)	26.00	14.00	33.00	87.00
GGT (U/L)	40.00	17.00	47.00	110.00
Total cholesterol (mmol/L)	6.90	5.40	6.10	6.36
Triglyceride (mmol/L)	1.60	2.80	1.88	4.97

HDL-c (mmol/L)	1.00	1.00	0.75	1.14
LDL-c (mmol/L)	4.40	3.70	3.97	4.23
LDL-c month 1 (mmol/L)	3.06	3.08	2.85	4.00
LDL-c month 2 (mmol/L)	2.86	2.88	2.65	3.81
LDL-c month 3 (mmol/L)	2.46	2.48	2.25	3.61
SGOT month 2 (U/L)	30.00	18.00	39.00	129.00
SGAT month 2 (U/L)	27.00	15.00	34.00	88.00
Muscle pain	No	No	No	No
Digestive disorder	No	No	No	No
LDLR gene variant	Yes	Yes	Yes	Yes
Variant	c.664T>C	c.1060+10G>C	c.1060+10G>C	c.1060+10G>C

Table S2. Summary of the gene variants found in this study.

Gene	Variant location	Nucleotide substitution	Amino acid alterations	Types of Variant	Database ID	dbSNP ID	Clinical classification	In silico pathogenic analysis			Number of index case	Number of relative
								Polyphen-2 HumVar	Polyphen-2 HumDiv	Mutation Taster		
LDLR	Exon 4	c.664T>C	p.Cys222Arg	Missense	LDLR_000707	rs577934998	Likely pathogenic	Probably damaging, 1	Probably damaging, 1	Disease causing (prob: 1)	1	38
LDLR	Intron 7	c.1060+10G>C		Splice site	LDLR_000478	rs12710260	Benign	-	-	-	3	57

Table S3. Baseline characteristics of patients with *LDLR* gene variant.

Pedigree	Age	Gender	BMI	History of MI	Hypertension	Diabetes	Increased total cholesterol	Increased triglyceride	Decrease HDL-c	Increased LDL-c	Dyslipidemia	*LDLR* Variant
1	72	female	24.7	Yes	Yes	Yes	Yes	Yes	No	Yes	Yes	c.664T>C
1	45	male	23.1	Yes	Yes	No	Yes	No	No	Yes	Yes	c.664T>C
1	15	female	18.9	No	No	No	No	No	No	Yes	Yes	c.664T>C
1	44	male	24.9	No	No	No	Yes	No	No	Yes	Yes	c.664T>C
1	18	male	23.7	No	No	No	No	No	No	No	No	c.664T>C
1	67	female	21.4	No	No	No	Yes	Yes	Yes	Yes	Yes	c.664T>C
1	35	female	20.8	No	No	No	Yes	Yes	No	Yes	Yes	c.664T>C
1	65	female	24.3	Yes	No	No	Yes	No	No	Yes	Yes	c.664T>C
1	45	female	19.0	No	No	No	Yes	No	No	Yes	Yes	c.664T>C
1	21	male	24.2	No	No	No	No	No	No	Yes	Yes	c.664T>C
1	61	female	20.8	No	No	Yes	Yes	Yes	No	Yes	Yes	c.664T>C
1	38	male	23.9	No	No	Yes	Yes	Yes	Yes	Yes	Yes	c.664T>C
1	7	male	21.0	No	No	No	No	No	No	No	No	c.664T>C
1	38	male	24.6	No	Yes	Yes	Yes	Yes	No	Yes	Yes	c.664T>C
1	53	male	23.6	No	Yes	Yes	Yes	No	No	Yes	Yes	c.664T>C
1	32	female	21.1	No	No	No	Yes	No	No	Yes	Yes	c.664T>C
1	29	male	23.2	No	No	No	No	No	No	Yes	Yes	c.664T>C
2	68	female	27.6	No	Yes	No	No	Yes	Yes	No	Yes	c.1060+10G>C
2	51	male	24.6	No	No	No	No	Yes	No	Yes	Yes	c.1060+10G>C
2	32	male	24.3	No	No	No	No	No	No	Yes	Yes	c.1060+10G>C
2	48	female	25.3	No	No	No	Yes	No	No	Yes	Yes	c.1060+10G>C
2	15	female	21.9	No	No	No	No	No	No	No	No	c.1060+10G>C
2	44	male	24.9	No	No	No	No	No	No	No	No	c.1060+10G>C
2	12	male	21.4	No	No	No	No	No	No	No	No	c.1060+10G>C
2	10	male	17.9	No	No	No	No	No	No	No	No	c.1060+10G>C
2	40	female	22.1	No	No	No	No	No	No	Yes	Yes	c.1060+10G>C
2	12	male	19.4	No	No	No	No	Yes	No	No	Yes	c.1060+10G>C

2	63	male	22.9	Yes	No	No	No	Yes	Yes	Yes	Yes	c.1060+10G>C
2	12	female	19.1	No	No	No	No	Yes	Yes	No	Yes	c.1060+10G>C
2	55	female	24.0	Yes	No	No	Yes	Yes	No	Yes	Yes	c.1060+10G>C
2	36	female	25.0	No	No	No	No	Yes	Yes	Yes	Yes	c.1060+10G>C
3	57	female	24.7	Yes	Yes	No	Yes	Yes	Yes	Yes	Yes	c.1060+10G>C
3	40	male	23.7	No	No	No	Yes	Yes	No	Yes	Yes	c.1060+10G>C
3	17	male	22.5	No	No	No	No	Yes	No	No	Yes	c.1060+10G>C
3	12	male	20.8	No	No	No	No	No	No	No	No	c.1060+10G>C
3	36	female	20.8	No	No	No	No	Yes	No	No	Yes	c.1060+10G>C
3	10	male	20.2	No	No	No	No	Yes	No	No	Yes	c.1060+10G>C
3	8	male	18.4	No	No	No	No	No	No	No	No	c.1060+10G>C
3	34	male	25.2	No	No	No	No	No	No	No	No	c.1060+10G>C
3	8	female	18.6	No	No	No	No	No	No	No	No	c.1060+10G>C
3	31	male	23.9	No	No	No	No	No	No	Yes	Yes	c.1060+10G>C
4	72	male	23.8	No	No	No	No	Yes	No	Yes	Yes	c.1060+10G>C
4	49	male	23.5	No	No	No	Yes	Yes	Yes	Yes	Yes	c.1060+10G>C
4	22	female	21.8	No	No	No	No	No	No	Yes	Yes	c.1060+10G>C
4	16	male	22.1	No	No	No	No	No	No	No	No	c.1060+10G>C
4	47	male	25.8	Yes	No	No	Yes	Yes	No	Yes	Yes	c.1060+10G>C
4	12	female	19.3	No	No	No	No	Yes	Yes	No	Yes	c.1060+10G>C
4	45	female	21.2	No	No	No	Yes	Yes	No	Yes	Yes	c.1060+10G>C
4	10	male	18.1	No	No	No	No	No	No	No	No	c.1060+10G>C

Table S4. Characteristics of the patients included in the lipid-lowering cohort.

	Total group (n=47)	Variant-positive (n=30)	Variant-negative (n=17)
Age (Mediann IQR) (years)	44 (37, 53)	45 (34, 56)	43 (39, 52)
Male (Yes) (n)	24	15 (50%)	9 (53%)
Pedigree (n)			
1	24	15 (50%)	9 (53%)
2	11	7 (23%)	4 (24%)
3	6	3 (10%)	3 (18%)
4	6	5 (17%)	1 (6%)
Smoking (yes) (n)	8	5 (17%)	3 (18%)
Hypertension (yes) (n)	6	5 (17%)	1 (6%)
Diabetes (yes) (n)	7	5 (17%)	2 (12%)
History of MI (yes) (n)	8	7 (27%)	1 (6%)
BMI (Mean±SD) (kg/m^2)	22.9±1.84	23.2±1.81	22.5±1.86

Table S5. Primer sequences for PCR amplification.

Primer name	Primer sequence (5' – 3')	Length (bp)	Region
LDLR-c1F	TCCCCCTGCTAGAAACCTCA	313	Exon 1
LDLR-g1R	ACAAGTCTCCCAGGGATGGA		
LDLR-g2F	TGGGTTCCTTCTTTGTGTCC	606	Exon 2
LDLR-g2R	TGGCTTTAAGGAAAGCTCCA		
LDLR-g3F	TATTGGCCAGGCTGGTCTTG	418	Exon 3
LDLR-g3R	GCGGAAGAGGCTTGGTATGA		
LDLR-g4F	GGCTATAGAATGGGCTGGTG	539	Exon 4
LDLR-g4R	GGCACCTAAATCACTGCATG		
LDLR-g5F	GGGCTCAAGCAATCCTCCTG	332	Exon 5
LDLR-g5R	CACAAATCATTTGCAAGCAG		
LDLR-g6F	CTGAGGCTCAGACACACCTG	293	Exon 6
LDLR-g6R	AAAGGAGCCCTTTGCACAGG		
LDLR-g7F	GAGGTGGAGGTTGTAATGAG	287	Exon 7
LDLR-g7R	ACTGAGGCATGAGGGGTTTG		
LDLR-g8F	CGCTCCGTCTCTAGCCATTG	288	Exon 8
LDLR-g8R	AGGGGATATGAGTCTGTGCA		
LDLR-g9F	ACCCTGCAGGATGACACAAG	655	Exons 9-10
LDLR-g10R2	AGCCCACTAACCAGTTCCTG		
LDLR-g11F	GACTATTTCCCAAGCCTGAG	1140	Exons 11-12
LDLR-g12R	AGGTCTAAGACCTCCTCCTA		
LDLR-g13F	CTCATCCCAGTGTTTAACGG	599	Exons 13-14

LDLR-g14R	TCCCGACTCATGAGTCCTTA		
LDLR-g15F	TCCTGGACTCACTCAAGAGA	429	Exon 15
LDLR-g15R	AGTGAGAGAAGGTCAGCAAG		
LDLR-g16F	CAAGTGTCCAGGGAGATGTG	263	Exon 16
LDLR-g16R	GCCAGGCACGAGGTCACATA		
LDLR-g17F	TTTTCACTCCAGCCACGGAG	348	Exon 17
LDLR-g17R	ATGCTGTCCTCGATCTGGAG		
LDLR-g18F	GCCCAGGAGGTGAGAAGTAG	184	Exon 18
LDLR-18R	AAAACAAGGCCGGCGAGGTC		
